# Effect of administration routes on the efficacy of human umbilical cord mesenchymal stem cells in type 2 diabetic rats

**DOI:** 10.3389/fendo.2025.1536655

**Published:** 2025-03-21

**Authors:** Qiqiang Tao, Youzhi Wu, Huiwen Pang, Pinglei Lv, Wenrui Li, Xuqiang Nie, Felicity Y. Han

**Affiliations:** ^1^ Hainan Beautech Stem Cell Anti-Aging Hospital, Qionghai, Hainan, China; ^2^ Australian Institute for Bioengineering and Nanotechnology, The University of Queensland, Brisbane, QLD, Australia; ^3^ College of Pharmacy, Zunyi Medical University, Zunyi, China; ^4^ Key Lab of the Basic Pharmacology of the Ministry of Education & Joint International Research Laboratory of Ethnomedicine of Ministry of Education, Zunyi Medical University, Zunyi, China

**Keywords:** umbilical cord mesenchymal stem cells (UCMSCS), type 2 diabetes mellitus (T2DM), administration routes, insulin, liver function

## Abstract

**Background:**

Human umbilical cord mesenchymal stem cells (UCMSCs) are being investigated in various clinical trials for different conditions, including type 2 diabetes mellitus (T2DM). However, there is limited research on the optimal injection routes for UCMSCs in T2DM, particularly intravenous injection.

**Objective:**

The objective of this study aims to investigate the efficacy of four different administration routes of UCMSCs in treating T2DM rats, including pancreas injection (DP), tail vein injection (DT), intraperitoneal injection (DI), and dorsal pancreatic artery injection (DPA).

**Results:**

After two weeks of UCMSCs treatment, the fasting blood glucose levels in the DT group decreased significantly. The oral glucose tolerance test (OGTT) levels and the islet structure in the DT group almost recovered to normal. The contents of C-P and GLP-1 in serum increased significantly in all treatment groups, while the levels of INS, TNF-α, IL-6, IL-1β, IAA, and GSP decreased significantly. These improvements were further observed after four weeks of UCMSCs treatment. Histological analysis confirmed the progression of pancreatic recovery in all treatment groups, with the DT group showing the most significant improvement, correlating with the observed efficacy. Immunohistochemistry results further demonstrated increased insulin and PDX-1 expression, along with reduced glucagon levels in UCMSCs-treated rats. Additionally, liver and kidney function significantly improved across all treatment groups, with the DT group showing the best outcomes.

**Conclusion:**

Overall, these findings suggest that the administration route significantly affected the efficacy of UCMSCs in treating T2DM, with tail vein injection showing the most effective results.

## 1 Introduction

Type 2 diabetes mellitus (T2DM) is a chronic, serious disease characterized by an unbalance of carbohydrate, lipid, and protein metabolism in the blood, along with insulin resistance, and inadequate insulin production (1, 2). According to the World Health Organization (WHO) database, the prevalence of diabetes for all ages worldwide was estimated to be 2.8% in 2000 and 4.4% in 2030 respectively (3). The cost of T2DM in individual, societal, and national aspects is shocking and drives national departments and academia to pay attention to primary prevention and treatment (4). Current treatments, including metformin, sulfonylureas, and insulin therapy, primarily manage symptoms but fail to address the underlying pathophysiology or reverse disease progression, highlighting the necessity for novel regenerative approaches (5, 6).

In recent years, significant progress has been made in therapeutic research for diabetes, with stem cell therapy which is emerging as a promising cure (7, 8). Among various stem cells, the umbilical cord mesenchymal stem cells (UCMSCs) have garnered increasing attention due to their remarkable biological characteristics, including self-proliferation, multilineage differentiation, and immunomodulatory properties (9). Compared to other stem cell sources, UCMSCs are derived from non-controversial, non-invasive sources, exhibit low immunogenicity, and possess a high capacity for tissue repair and regeneration, making them ideal candidates for clinical applications (10, 11). UCMSCs have shown the ability to modulate immune responses, reduce inflammation, and promote tissue regeneration (10, 11), which are critical for addressing the complex pathophysiology of diabetes. Currently, human UCMSCs are implemented and under investigation for various clinical trials in different conditions and stages, including neurological diseases (12), autoimmune diseases (13), endocrine system diseases (14), etc., showing the safety of transplantation/injection and improvements in clinical symptoms (7). In ongoing clinical trials, the main routes of delivery are intrathecal, intravenous, and local delivery (7). There were some studies indicating that injection of human UCMSCs via intravenous (tail vein) has similar therapeutic efficacy compared with intrahepatic injection in acute liver failure rat models (15), and other clinical studies via the pancreatic artery or pancreaticoduodenal artery (16). Several studies, including clinical trials, have investigated the safety and efficacy of human UCMSCs therapy for type 1 diabetes and T2DM (17–19). For instance, a randomized controlled trial by Zang et al. reported that intravenous UCMSCs administration in T2DM patients significantly improved β-cell function and reduced HbA1c levels over 12 months (19). Despite these advances, there is limited research on the optimal injection routes for human UCMSCs in T2DM, particularly intravenous injection.

In this study, the efficacy of UCMSCs in the treatment of T2DM was investigated in T2DM rats including four distinct injection routes, tail vein, pancreatic, intraperitoneal, and pancreatic artery intravenous with tail vein injection of saline and T2DM rats without treatment as another control groups. Our study evaluated longitudinal changes in glucose homeostasis (FBG, OGTT), inflammatory cytokines (TNF-α, IL-6, IL-1β), pancreatic histopathology, and renal/liver function over four weeks. By examining the outcomes and the differences among these four administration routes, we seek to establish a solid theoretical foundation for the clinical application of human UCMSCs transplantation in treating T2DM.

## 2 Experimental section

### 2.1 Materials

Human UCMSCs were obtained from Hainan Beautech Stem Cell Anti-aging Hospital Co.Ltd, Hainan, China. Streptozotocin (STZ, S8050), glucose anhydrous (G8150), rat tumor necrosis factor α (TNF-α) ELISA kits (SEKR0009), rat interleukin 6 (IL-6) ELISA kits (SEKR0005), rat interleukin 1β (IL-1β) ELISA kits (SEKR0002), paraffin with ceresin (YA0011), neutral balsam (G8590), total cholesterol content assay kits (BC1980), and triglyceride content assay kits (BC0620) were purchased from Beijing Solarbio Science & Technology Co., Ltd., Beijing, China. Rat C-peptide (C-P) ELISA kits (JL20784), rat insulin (INS) ELISA kits (JL10692), rat glycated serum protein (GSP) ELISA kits (JL21292), rat glucagon-like peptide 1 (GLP1) ELISA kits (JL12394), rat high density lipoprotein (HDL) ELISA kit (JL13845), rat low density lipoprotein (LDL) ELISA kit (JL13846), and rat glycated hemoglobin (HbA1c) ELISA kit (JL21291) were purchased from Shanghai Jianglai Biotechnology Co., Ltd., Shanghai, China. Rat insulin autoantibodies (IAA) ELISA kits (ml003344), rat urea nitrogen (BUN) content kits (100T/96S), were from Shanghai Enzyme-linked Biotechnology Co., Ltd., Shanghai, China. The high-fat diets were from SPF (Beijing) Biotechnology Co., Ltd., Beijing, China. Blood glucose test strips were purchased from the Sinocare Biosensor Co., Ltd. Changsha, China. Ethanol absolutes were purchased from Sinopharm Chemical Reagent Co., Ltd., Shanghai, China. Creatinine colorimetric (CCr) assay kits (E-BC-K188-M) and rat microalbuminuria (MAU) ELISA kits (E-EL-R0025c) were purchased from Elabscience Biotechnology Co., Ltd. Wuhan, China. e-cadherin antibody (sc-8426) was obtained from Santa Cruz Biotechnology, Inc. Shanghai, China. Recombinant anti-insulin (INS) antibody (ab181547), recombinant anti-glucagon (GLU) antibody (ab92517), recombinant anti- pancreatic and duodenal homeobox 1 (PDX-1) antibody (ab219207), recombinant anti-collagen I (Col-I) antibody (ab270993), goat anti-rabbit IgG H&L (ab6721), anti-alpha smooth muscle actin (α-SMA) antibody (ab7817), goat anti-mouse IgG H&L (ab6789) were purchased from ABcam, UK.

### 2.2 Induction of T2DM in rats by high-fat diets and STZ

Male Sprague-Dawley (SD) rats (150-180 g) were used for all experiments to avoid any possible impact of the estrous cycle on the study outcomes. Animals were purchased from Shanghai Silaike Experiment Animal Co., Ltd. The rats were housed in groups of up to three under standard conditions in cages with recycled paper bedding in a 12/12 h light/dark cycle facility. Rats were free of food and water and allowed one week of acclimatization to the environment prior to the commencement of any procedures. Animal experiments were performed in accordance with approval from the Hainan Beautech Stem Cell Anti-Aging Hospital Ethics Committee, Hainan, China (the approval number is S230701).

T2DM rats were induced by feeding on a high-fat diet (67.5% conventional feed, 10% lard, 20% sucrose, and 2.5% cholesterol) and intraperitoneal (i.p.) injection of STZ according to the previous study (20). Briefly, rats were fed the high-fat diet for 4 weeks. The body weights of rats were recorded once a week. Following this initial 4-week period, the diet was switched to a clean-grade standard feed to sustain the experimental conditions, and rats received STZ intraperitoneally (20 mg/kg, dissolved in 0.1 mM citrate buffer (pH = 4.2)) daily for 3 days. The establishment and stability of T2DM in rats were tracked weekly. Rats with glucose levels 12.2–13.9 mmol/L in 4 weeks post-STZ injection will be considered diabetic and used for the designed study (21).

### 2.3 Study design

**Figure 1:**
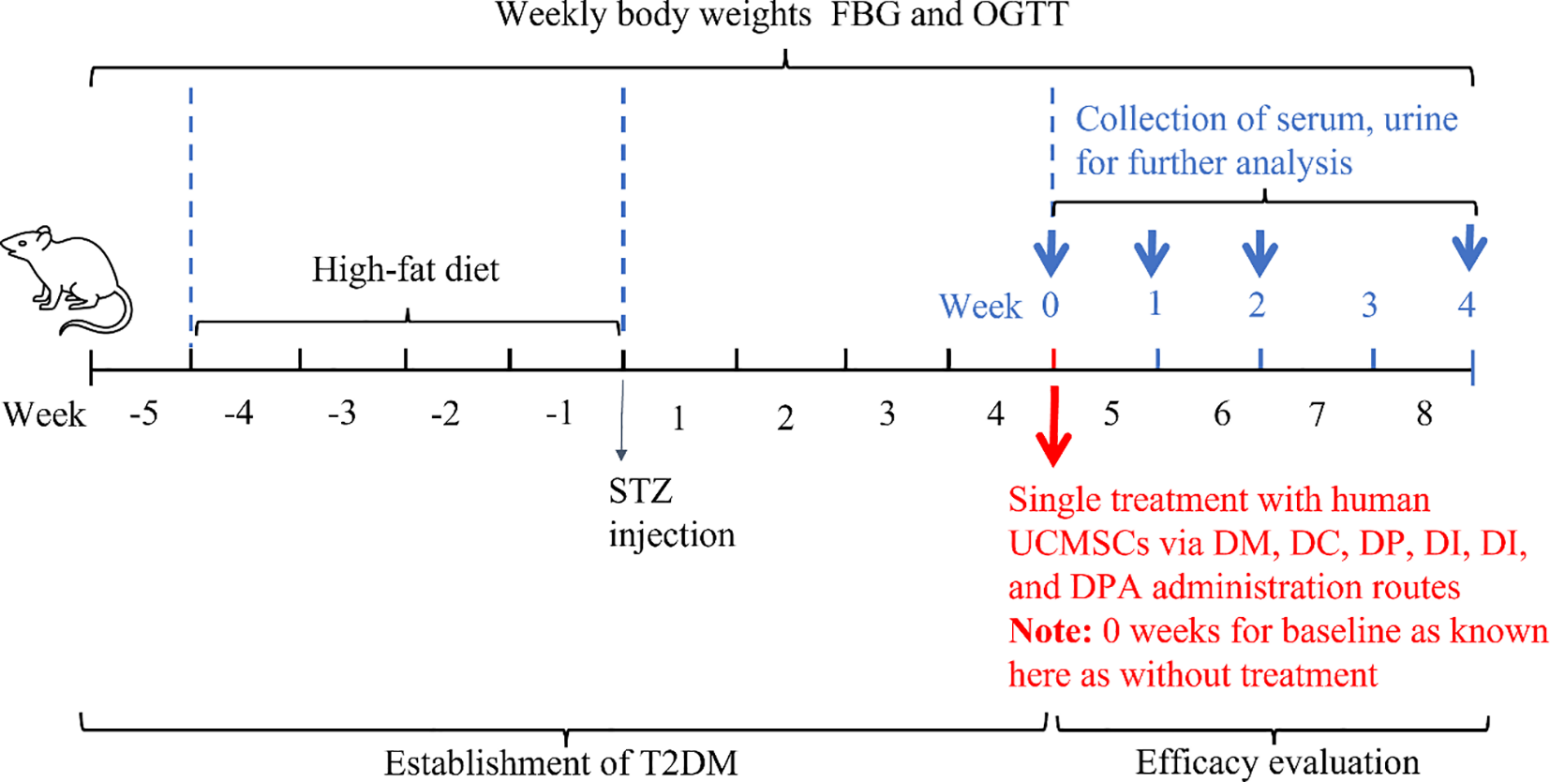
Schematic overview of the experimental protocol for efficacy evaluation of human UCMSCs in T2DM rats by comparing four administration routes.

The objective of this study was to compare the efficacy of human UCMSCs in T2DM rats by different administration routes in a blinded manner. A schematic diagram of the study procedures is shown in **Figure 1**. After confirming the establishment of T2DM, rats were randomly divided into six groups as detailed in **Table 1**. A single dose of human UCMSCs was given to T2DM rats by four different administration routes with two control groups. The four administration routes are T2DM rats treated with human UCMSCs (1×10^6^) by tail vein injection (DT), pancreas injection (DP), intraperitoneal injection (DI) and dorsal pancreatic artery injection (DPA). The two control groups are T2DM rats without treatment (DT), and T2DM rats treated with 0.2 ml saline via tail vein injection (DC).

**Table 1:** Study design (Type 2 diabetes mellitus (T2DM) rats, n=12).

Groups	Treatment	Administration route
DM	No	N/A
DC	0.2 ml saline	Tail vein injection
DP	0.2 ml human UCMSCs (1 × 10^6^ cells)	Pancreas injection
DT		Tail vein injection
DI		Intraperitoneal injection
DPA		Dorsal pancreatic artery injection

### 2.4 FBG measurement and OGTT

Before the test, all experimental rats underwent a 12-h fasting period. On the day of the test, the rats were weighed, and FBG levels were measured using a blood glucose meter (Sinocare, Hunan, China). Subsequently, a glucose solution (2 g/kg) was administered orally to the rats, and blood samples were collected to measure the OGTT (22).

### 2.5 Serum and urine analysis

At the end of the experiment (4 weeks after human UCMSCs or saline injection), rats were euthanized, and blood, urine and tissues were collected after the final measurement of FBG and OGTT. Tissues were fixed with 4% PFA for histology study. The content of biomarkers in serum and urine samples was measured by the biochemical rats ELISA or assay kits (C-P, INS, GSP, GLP1, HDL, LDL, HbA1c, IAA, BUN, CCr, MAU) following the manufacturer manual (23).

### 2.6 Histological analysis

The fixed pancreas and kidney tissues were embedded in paraffin and 5 µm serial sections were subjected to histology analysis (H&E, immunohistochemistry and TUNEL staining) according to the previous study (24, 25), and. Immunohistochemistry of pancreas and kidney tissues was performed according to studies by others (26). Briefly, sections were treated with EDTA to the recovery of antigen after dewaxing, then blocked with 10% (v/v) normal goat serum for 20 min at 37°C, followed by incubation with the primary antibody (concentrations: INS 1:64000, GLU 1:8000, PDX-1 1:500, e-cadherin 1:50, Col-l 1:500, α-SMA 0.034 µg/ml), or phosphate-buffered saline as the negative control, overnight at 2-4°C overnight. The next day, sections were incubated with a secondary antibody of horseradish peroxidase-labelled goat anti-rabbit IgG for 30 min at 37°C. Then after washing with PBS, the sections were incubated with the 3,3′-diaminobenzidine (DAB) solution. Finally, sections were hematoxylin counterstained after washing with running water for 20 min, dehydrated, and sealed for imaging.

### 2.7 Statistical analysis and data availability

The data are presented as mean (± SD). The t-test was used to compare the means between treatment groups and controls and Cohen’s d value was used to identify the effect size for measuring the difference between treatment groups and control groups (27). The one-way ANOVA followed by Tukey’s multiple comparison test was used to analyze the between-group differences. GraphPad P v10 (GraphPad Software, La Jolla, United States) was used for statistical analyses. The statistical significance criterion was P ≤ 0.05. Numerical data for this study have been deposited in an open data repository for public access: http://doi.org/10.5281/zenodo.14955051.

## 3 Results

### 3.1 Establishment of T2DM

**Figure 2:**
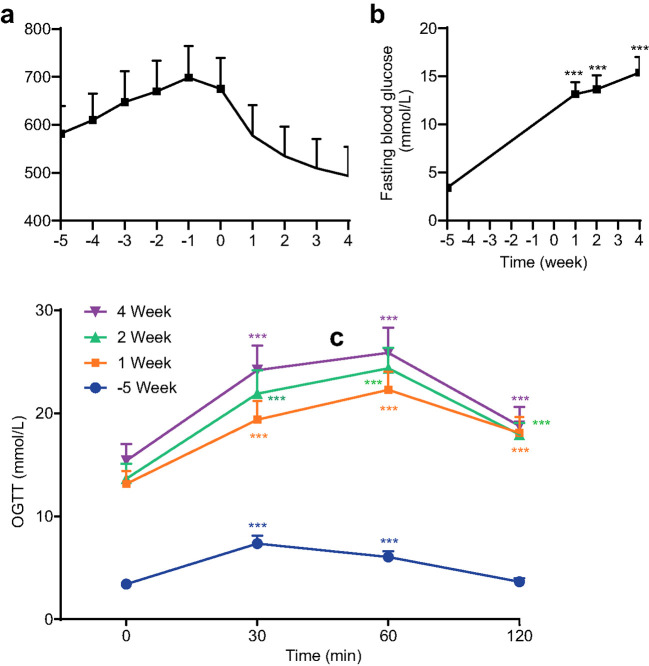
Body weight **(a)**, fasting blood glucose (FBG) levels **(b)**, and oral glucose tolerance tests (OGTT) levels **(c)** of all rats for all groups before treatment (*** P<0.001 vs baseline at week -5 or 0 min, n=72, with Cohen’s d > 0.8 indicating a large effect size).

T2DM was induced successfully in all rats as shown in **Figure 2**. As shown in **Figure 2A**, the body weight of all mice increased significantly (P ≤ 0.01) following one week of a high-fat diet and consistently increased till STZ injection. Body weight was then decreased 1 week (P ≤ 0.001) after STZ injection significantly and consistently decreased in 2 weeks, 3 weeks, and 4 weeks. In comparison with the baseline (before the high-fat diet, week -5) FBG levels increased substantially and consistently following STZ injection (**Figure 2B**). The OGTT in **Figure 2C** indicated the abnormal glucose tolerance of rats after 1 week of STZ injection for T2DM model induction.

### 3.2 FBG levels after human UCMSCs treatment

**Figure 3:**
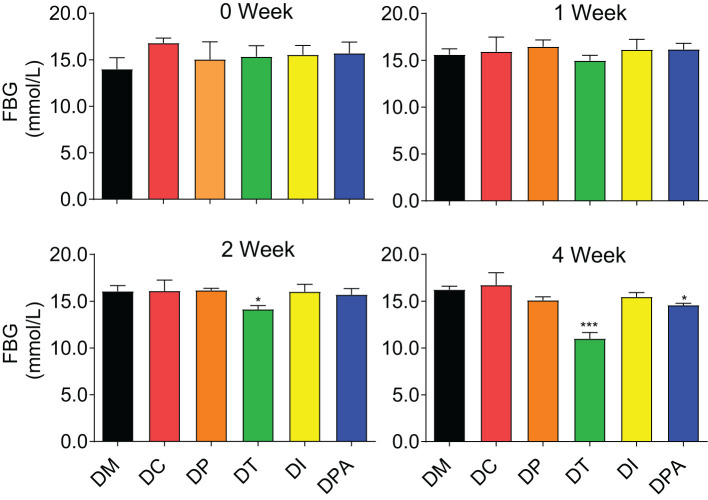
Fasting blood glucose (FBG) levels at 0, 1, 2, and 4 weeks after human UCMSCs treatment (* P<0.05, *** P<0.001 vs DM Group with Cohen’s d > 0.8 indicating a large effect size).

T2DM rats were treated with human UCMSCs (single injection) compared to saline control and an additional group of T2DM without treatment. As shown in **Figure 3**, there were no improvements in all groups after receiving treatment of UCMSCs in one week. After two weeks of the treatment, only the DT group showed a significant decrease in FBG. After 4 weeks of human UCMSCs treatment, FBG in DT (P<0.001) and DPA (P<0.05) groups decreased significantly, and other groups did not show significant changes in FBG.

### 3.3 OGTT after human UCMSCs treatment

**Figure 4:**
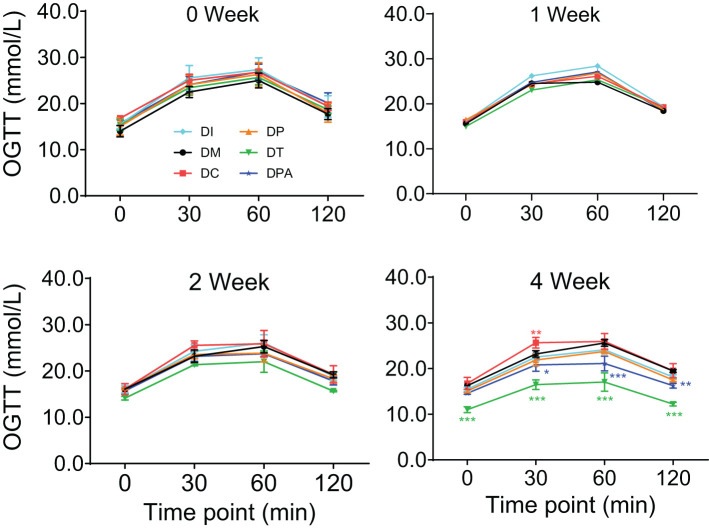
Changes in OGTT levels in 0, 1, 2, and 4 week after human UCMSCs treatment (*P<0.05, **P<0.01, ***P<0.001 vs DM Group with Cohen’s d > 0.8 indicating a large effect size).

To further investigate the therapeutic effect, the OGTT levels were evaluated (**Figure 4**). There were no significant changes in OGTT levels observed between each group in 1 week after human UCMSCs injection. The OGTT levels in the DT group showed a recovery in 2 weeks after treatment, but no significant difference in the other groups. After four weeks of treatment of UCMSCs, the OGTT levels were significantly improved in both DT (P<0.001) and DPA (P<0.01) groups but not in the remaining groups.

### 3.4 Glucose metabolism and metabolomic changes in response to the human UCMSCs treatment

**Figure 5:**
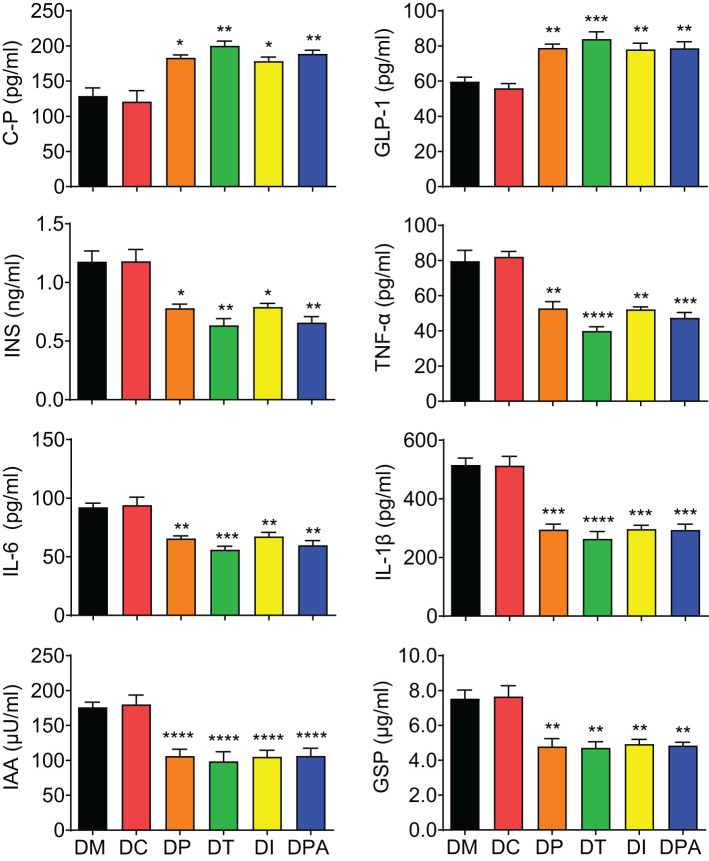
Serum biomarker levels of each group at 4 weeks post-treatment. C-P: C-peptide, INS: Insulin, TNF-α: Tumor necrosis factor-alpha, IL-6: Interleukin 6, IL-1β: Interleukin-1 beta, IAA: Insulin autoantibodies, GSP: Glycated serum protein, and GLP-1: Glucagon like peptide-1. *P<0.05, **P<0.01, ***P<0.001, ****P<0.0001 vs DM Group with Cohen’s d > 0.8 indicating a large effect size.

The glycometabolism biomarkers in serum were further analyzed. There was a significant elevation (P<0.05) in the levels of C-P and GLP-1, and a marked reduction (P<0.05) in INS, TNF-α, IL-6, IL-1β, IAA, and GSP across all treatment groups after 4 weeks of treatment (**Figure 5**). There were no significant changes in these biomarkers were observed across the various treatment groups compared to the DM group before treatment (0 weeks) and 1-week post-treatment (**Supplementary Figure S1A**). However, after 2 weeks of treatment, C-P and GLP-1 levels were significantly increased (P<0.05) in all treatment groups, and INS, TNF-α, IL-6, IL-1β, IAA, and GSP levels showed a significant decrease (P<0.05) (**Supplementary Figure S1B**). No substantial changes were observed in any biomarkers tested in the DC group compared to the DM group.

### 3.5 Liver and kidney function after the human UCMSCs treatment

To comprehensively assess the physiological effects following human UCMSCs therapy, the impact on liver and kidney function was evaluated. There was a marked increase in HDL, UCr, and CCr, and a significant decrease in TC, TG, LDL, HbA1c, BUN, SCr, and MAU in all human UCMSCs treatment groups compared to DM rats, but not in the saline-treated DC group by week 2 of the treatment (**Supplementary Figures S2, S3**). The DT group showed the best outcomes. No significant changes were observed in all treatment groups before treatment (0 weeks) and 1 week post-treatment.

Additionally, both DM and DC groups exhibited disrupted kidney structures post-treatment, which was characterized by renal corpuscular atrophy, hypertrophy of renal tubular epithelial cells, irregular tubular lumen morphology, and localized necrosis, with some necrotic areas showing calcium salt deposition (**Supplementary Figure S4**). Tubular vacuolar degeneration was also observed, indicated by pale-staining, loose cytoplasm containing multiple vacuoles in the DM and DC groups. In contrast, the DP, DI, and DPA groups showed gradual improvement over time, as evidenced by reduced renal corpuscular atrophy, alleviated tubular degeneration, and minimal calcium salt deposition. Notably, the DI group exhibited the most significant renal recovery, particularly at week 4, where glomeruli appeared nearly normal, renal tubular epithelial cell size returned to normal, the tubular lumen exhibited a regular shape, while vacuolar degeneration and calcium salt deposition were almost undetectable.

### 3.6 Pathological changes of the pancreas after the human UCMSCs treatment

**Figure 6:**
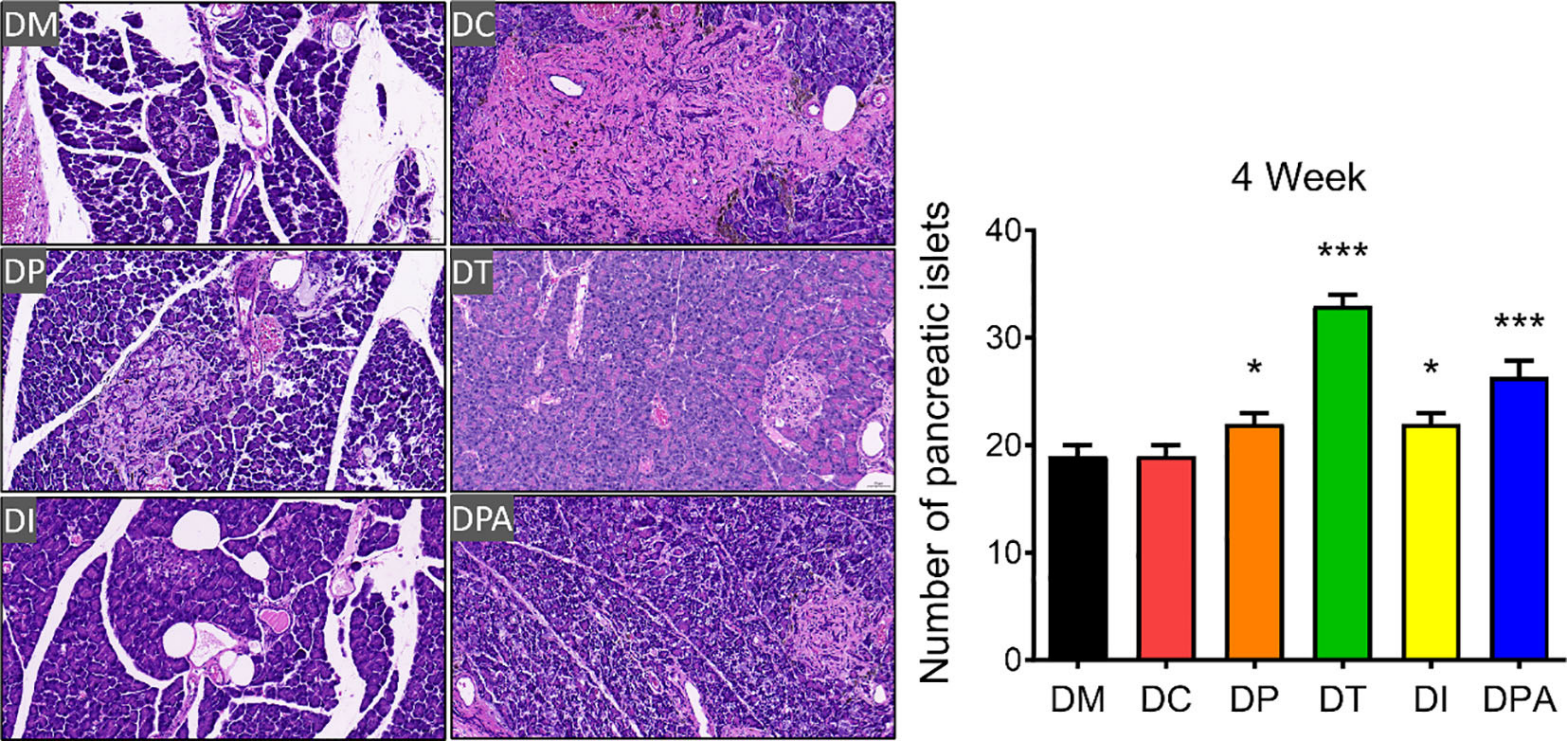
H&E staining of pancreatic tissues and the number of islets after 4 weeks of treatment (20×, n=3). *P<0.05, ***P<0.001, vs DM Group with Cohen’s d > 0.8 indicating a large effect size. At week 4, islet cell atrophy and vacuolation were significantly reduced in the DP, DT, DI, and DPA groups.

**Figure 7:**
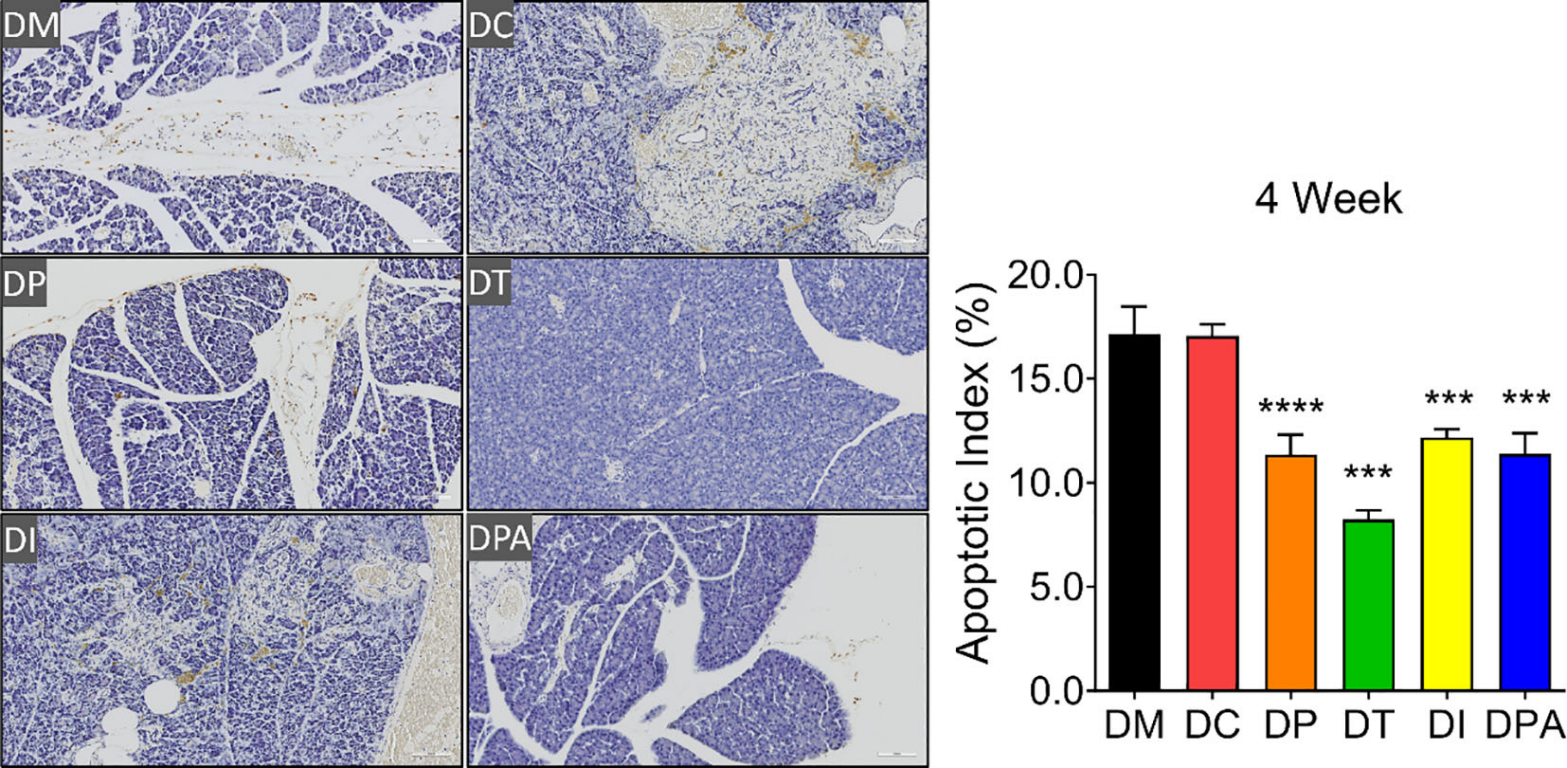
TUNEL staining of pancreatic tissues after 4 weeks of treatment (20×, n=3). *** P<0.001, **** P<0.0001 vs DM Group with Cohen’s d > 0.8 indicating a large effect size.

As shown in **Supplementary Figure S5**, H&E staining results showed that the pancreatic tissue structure in the DM and DC groups was severely damaged, characterized by islet cell atrophy and focal vacuolar changes. After 2 weeks of the treatment, compared to the DM and DC groups, the number of islets significantly increased in the DP, DT, DI, and DPA groups, with the DT group exhibiting the most pronounced increase (**Supplementary Figure S5**). At week 4, islet cell atrophy and vacuolation were markedly reduced, and the tissue structure showed significant improvement in all treatment groups. The islet morphology tended to recover, and the number of islets significantly increased (**Figure 6**). Notably, the DT group showed the best outcomes with relatively intact islet structures, indicating near-complete recovery. In addition, before treatment (0 weeks), irregularities in pancreatic tissue structure and signs of islet cell atrophy, coupled with localized vacuole-like alterations, were evident across all rat groups. There was no significant change after 1 week of treatment. The TUNEL staining results also showed that at weeks 2 and 4, islet cell apoptosis was significantly reduced in the DP, DI, and DPA groups, with the DT group exhibiting the most pronounced reduction (**Figure 7**; **Supplementary Figure S6**).

### 3.7 Immunohistochemistry analysis of pancreas and kidney tissues

**Figure 8:**
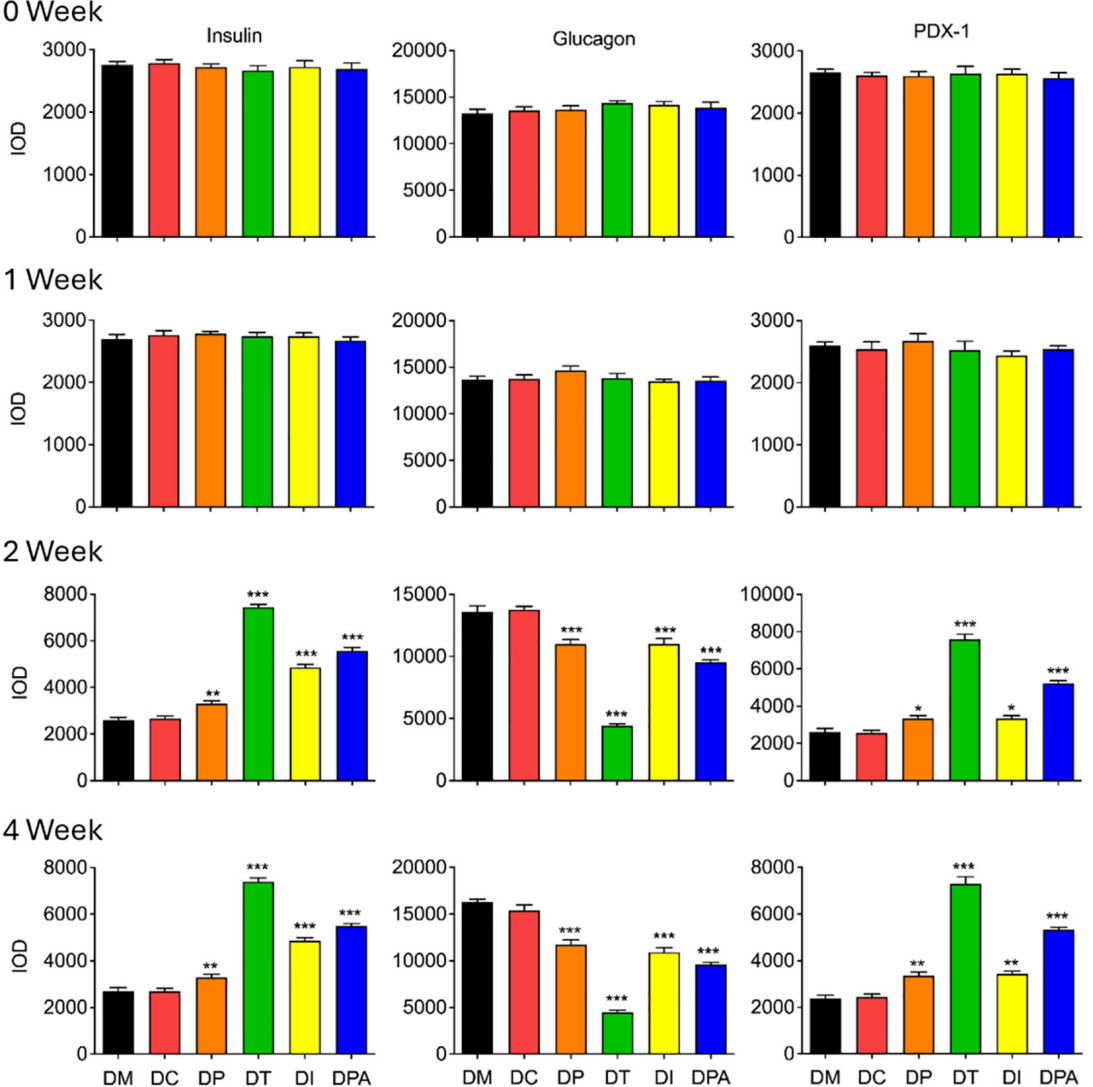
Expression of insulin, glucagon, and PDX-1 in pancreases after 0, 1, 2, and 4 weeks of human UCMSCs treatment (*P<0.05, **P<0.01 and ***P<0.001 vs DM Group, with Cohen’s d > 0.8 indicating a large effect size.). IOD: Integrated optical density, PDX-1: Pancreatic and duodenal homeobox 1.

To evaluate the treatment efficacy at the tissue level, insulin, glucagon, PDX-1 were further assessed in pancreatic and renal tissues by immunohistochemistry (28). There were no significant alterations in the mean expression levels of insulin, glucagon, and PDX-1 in the pancreatic tissue of rats when compared to the DM group in 0 and 1 week post-treatment (**Figure 8**; **Supplementary Figures S7A-C**). While in 2 and 4 weeks after treatment, the average expression of insulin, glucagon and PDX-1 in the pancreatic tissue of rats in the DC group did not change significantly, but the expression of glucagon in the pancreatic tissue of all human UCMSCs groups decreased significantly, and the expression of insulin and PDX-1 increased significantly during this period (**Figure 8**; **Supplementary Figures S7A-C**).

Consistently, there were no significant differences in the expression of e-cadherin, collagen-I, and α-SMA in renal tissue when comparing rats from the DM group in 0 and 1-week post-treatment (**Supplementary Figures S7D-F**, **S8**). Notably, after 2 and 4 weeks of treatment, there were no significant changes in renal tissue between rats in the DM and DC group, but the expression of collagen-I and α-SMA in all human UCMSCs treatment groups exhibited significant reductions, and the expression of e-cadherin displayed a substantial increase (**Supplementary Figures S7D-F**, **S8**).

## 4 Discussion

As expected, the efficacy of human UCMSCs is affected by the administration routes in treating type 2 diabetes in rats. Previous studies have demonstrated that MSCs have a natural homing ability to the damaged or inflamed tissues, including the pancreas in diabetic models (29–31). Although all treatment groups were effective to some extent, the intravenous (tail vein) injection (DT group) proved to be the most effective, followed by the DPA group. In two weeks post-treatment, the DT group rats exhibited a significant decrease in FBG levels and a concurrent improvement in OGTT levels (**Figures 3**, **4**). By four weeks, both the DT and DPA groups displayed substantial reductions in FBG levels and normalized OGTT levels, while no significant changes in FBG and OGTT were observed in the other groups. Similar therapeutic results have been observed in db/db mice (32). A single intravenous injection of UCMSCs in 7-week-old male db/db mice significantly reduced blood glucose levels within 7 days post-injection for up to 5 weeks alongside notable improvements in hepatic glucose and lipid metabolism (32).The primary mechanism was attributed to enhanced Akt phosphorylation, which ameliorated glucose and lipid metabolism dysfunction and reduced inflammation in the livers of db/db mice (32). In contrast, our findings reveal that 2 weeks after human UCMSCs treatment, serum levels of C-P and GLP-1 significantly increased across all four treatment groups. DT group demonstrated more significant change compared to the other three treatment groups (**Figure 5**). Previous studies indicated that decreased C-P was associated with an increased risk of T2DM in the general population (33). Similarly, GLP-1 plays another key role in the development of T2DM, including regulation of glucose homeostasis, insulin secretion, and alteration of insulin resistance (34). In agreement with our results, Zang et al. found that the C-P area under the curve improved after human UCMSCs intravenous infusion at 9 and 48 weeks in T2DM patients, and the C-P area under the curve could be an independent risk factor associated with efficacy in T2D undergoing human UCMSCs intervention (19).

A significant reduction in INS, TNF-α, IL-6, and IL-1β levels was observed in our study, indicating an improved diabetic state, diminished insulin demand, reduced insulin autoantibodies, and a more pronounced therapeutic effect in these groups after 4 weeks of human UCMSCs injection. Notably, the DT group showed the most significant improvements compared to the other three treatment groups. Notably, Sun et al. demonstrated that human UCMSCs could also inhibit the occurrence and development of T2DM by suppressing NLRP3-related proinflammatory cytokines (35). Previous studies on T2DM patients and T2DM mouse models have demonstrated poor glycemic control with significantly elevated levels of proinflammatory cytokines, including TNF-α, IL-6, and IL-1β (36, 37). Consequently, the reduction in anti-inflammatory cytokine levels suggests attenuation of *in vivo* inflammation. Some studies demonstrated the immunosuppressive properties of mesenchymal stem cells in the proliferation of T lymphocytes. This inhibition is expressed in the modulation of T cell metabolic pathways, fostering T cell tolerance, and promoting the expansion of regulatory T cell populations (38). Additionally, mesenchymal stem cells showed inhibitions to the proliferation of B cells and suppress various immune cell functions, including cytokine secretion and cytotoxicity of T and natural killer cells, B cell maturation, and antibody secretion (39, 40).

In addition, the liver and kidney functions were assessed through serum and urine analyses. In week 2, the four treatment groups exhibited a notable elevation in serum HDL levels and a significant reduction in TC, TG, LDL, and HbA1c levels compared to the two control (DM and DC) groups (**Supplementary Figures S2**, **S3**). Consistently, the DT group was better than other three treatment groups. The pancreas, adipose tissue, liver, and intestines as well as the kidneys also play a significant role in glycemic level of control, including renal gluconeogenesis and the reabsorption of glucose within the kidney (41). Previous correlation studies in T2DM patients indicated that significantly higher serum levels of TC, TG and LDL-C and significantly lower serum levels of HDL-C were found in T2DM patients, as well as strong correlations between HbA1c and TC, TG, and HDL-C (42). In agreement with others, our results suggest potential liver and kidney function and the T2DM condition recovery in all four treatment groups by 4 weeks post-treatment.

Furthermore, histopathological examination of pancreatic tissue revealed that all treatment groups exhibited reduced islet cell atrophy and vacuolization compared to control groups (**Figures 6**, **7**). These findings correlate with the improvements observed in biochemical indicators. It is speculated that the human UCMSCs intervention in T2DM may improve the symptoms of diabetes by modulating the immune system and reducing local inflammation (43). According to previous studies, expressions of insulin, glucagon, PDX1, e-cadherin, collagen-I, and α-SMA could demonstrate the development of T2DM conditions (44–46). Our data showed significant changes in these tested parameters after 2 and 4 weeks of treatment but not in the control groups (**Figure 8**; **Supplementary Figure S8**). Among these groups, the DT group had a more significant decrease in glucagon, collagen-I, α-SMA, and an increase in insulin, PDX-1, and e-cadherin. PDX-1 is the most critical and indispensable not only for the regulation of β-cells but also for the function of the pancreatic gene regulatory network (47). For, example, the normal function of the pancreatic cell lineage requires a high concentration of PDX-1 (48). The increase of PDX-1 was observed after 2 and 4 weeks in all four treatment groups. DT group had a more significant increase in the expression of PDX-1. E-cadherin at the surface of islet β-cells secretion is controlled by secretagogues including glucose (49). E-cadherin correlates with insulin and can serve as a surface marker of β-cell function (49). The increase of e-cadherin after 2 and 4 weeks of treatment likely indicated the meliorate of β-cell in the treatment groups, especially in the DT group. Glucagon is a key regulator of normal fuel metabolism, and both fasting and postprandial hyperglucagonemia make substantial contributions to the fasting hyperglycemia and postprandial glucose excursions that characterize T2DM (44). Patients with T2DM were observed with higher concentrations of glucagon (50). The decrease in glucagon suggests better control of insulin and the development of T2DM. Besides, the α-SMA is a protein expressed early in vascular smooth muscle cell differentiation, and the levels of α-SMA increase when vascular smooth muscle cells become quiescent state (51). The decrease of α-SMA indicated that the vascular smooth muscle cell is more active after 2 weeks post-treatment, addressing the recovery of renal functions with T2DM. On the one hand, collagen-I could improve the INS-1 cell growth and insulin biosynthesis (52). On the other hand, inhibition of collagen-I accumulation reduces glomerulosclerosis in diabetic nephropathy (53). The level of collagen-I was reduced after 2 weeks of post-treatment, indicating a better balance of collagen-I in treatment groups, especially in DT group rats.

Overall, our findings show that human UCMSCs treatment reduces pro-inflammatory cytokines and promotes histological recovery in pancreatic and hepatic tissues, supporting its therapeutic potential for T2DM. However, the proposed mechanisms, such as oxidative stress, related lipid metabolism and signaling pathways, require further validation. Future studies should include biodistribution analyses to track human UCMSCs homing and engraftment and explore the longer-term effects which will further clarify why tail vein injection appears most effective and strengthen our understanding of UCMSCs mechanisms.

## 5 Conclusion

The human UCMSCs (1×10^6^ cells) were effective in treating T2DM in rats across all treatment groups to some extent by a short term of 4 weeks post-treatment. Intravenous injection (DT Group) was the most effective among the tested routes followed by DPA Group. A significant decrease in FBG levels and a return toward normal levels in OGTT was observed in 2 weeks post-treatment. Changes in various biochemical markers supported the efficacy observed in FBG and OGTT levels. Moreover, examination of pancreatic and kidney tissues confirmed histological recovery in T2DM rats treated with UCMSCs, correlating with the observed efficacy. Future studies on extending the observation period, administering repeated infusions, and exploring different doses of UCMSCs may enhance the efficacy of treatment further.

## Data Availability

The datasets presented in this study can be found in online repositories. The names of the repository/repositories and accession number(s) can be found in the article/**Supplementary Material**.
