# Supplementary material for: Effect of administration routes on the efficacy of human umbilical cord mesenchymal stem cells in type 2 diabetic rats
Source: Front Endocrinol (Lausanne). 2025 Mar 21;16:1536655. doi: 10.3389/fendo.2025.1536655 (PMC11968364; doi:10.3389/fendo.2025.1536655)
Supplement: Supplementary file 1 [file DataSheet1.pdf]

Supplementary Materials

Effect of administration routes on the efficacy of human umbilical cord mesenchymal stem cells in type 2 diabetic rats

Qiqiang Tao[1]*, Youzhi Wu[2], Huiwen Pang[2], Pinglei Lv[1], Wenrui Li[1], Xuqiang Nie[2,3,4], Felicity Y. Han[2]*

[1] Hainan Beautech Stem Cell Anti-Aging Hospital, Hainan 571400, China.

[2] Australian Institute for Bioengineering and Nanotechnology, The University of Queensland, Brisbane, QLD 4072, Australia.

[3] College of Pharmacy, Zunyi Medical University, Zunyi 563006, China

[4] Key Lab of the Basic Pharmacology of the Ministry of Education & Joint International Research Laboratory of Ethnomedicine of Ministry of Education, Zunyi Medical University, Zunyi 563006, China

* Correspondences: taoqiqiang@beautech.net.cn; f.han@uq.edu.au

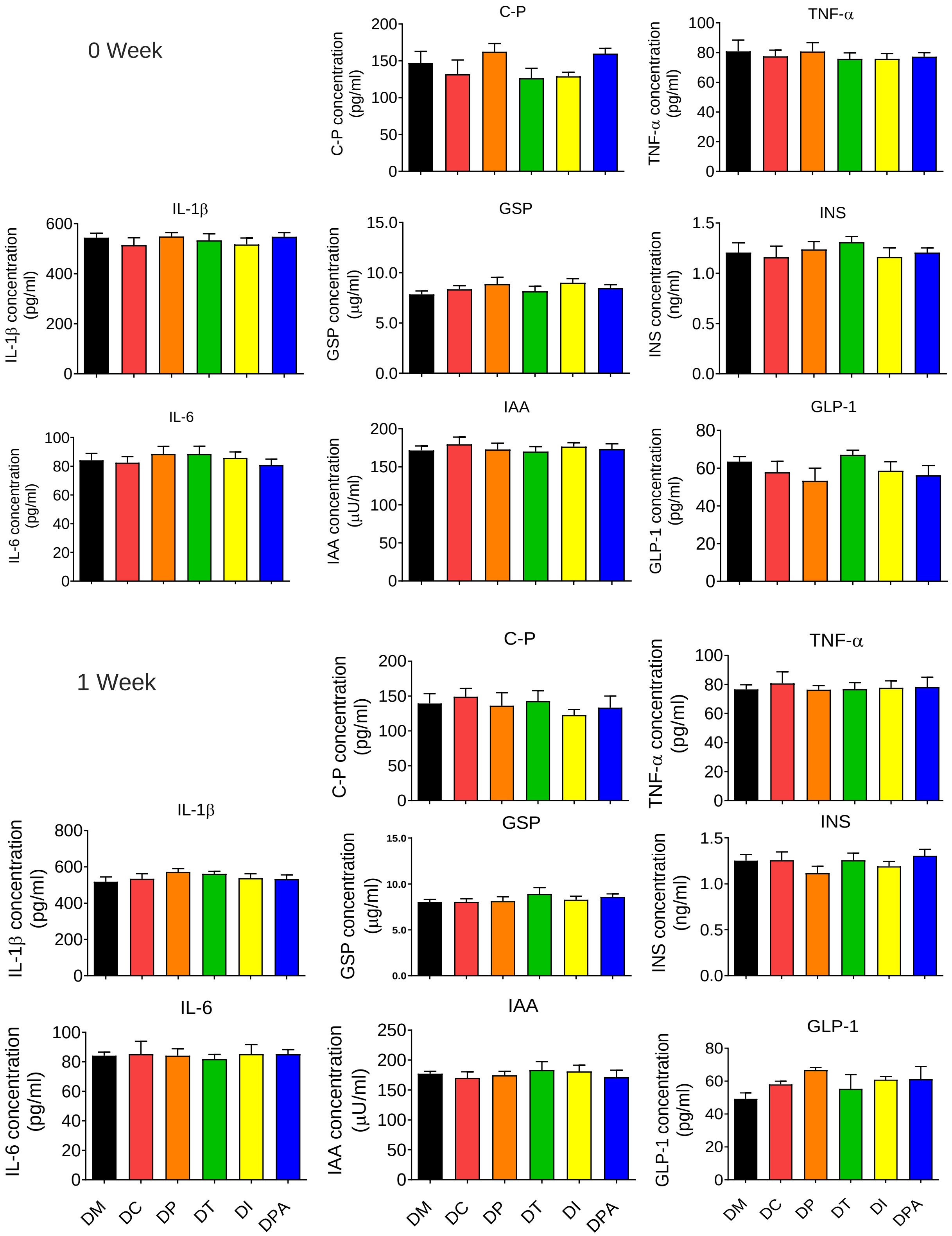

Figure S1a. Serum biomarker levels of each group at 0 and 1 weeks post-treatment.

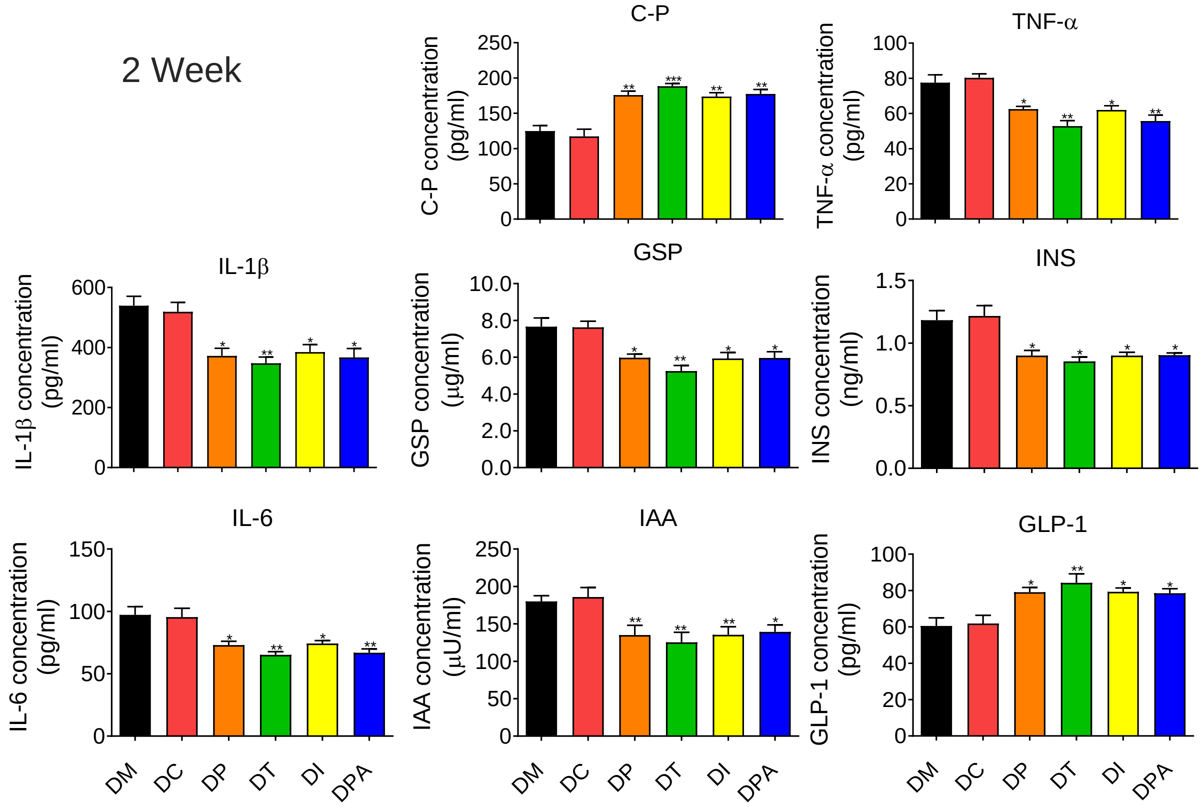

Figure S1b. Serum biomarker levels of each group at 2 weeks post-treatment.
C-P: C-peptide, INS: Insulin, TNF-α: Tumor Necrosis Factor-Alpha, IL-6: Interleukin 6, IL-1β: Interleukin-1 Beta, IAA: Insulin autoantibodies, GSP: Glycated serum protein, and GLP-1: Glucagon Like Peptide-1. DM: T2DM rats without treatment, DC: T2DM rats treated with 0.2 ml saline (Tail vein, negative control), DP: T2DM rats treated with 0.2 ml of human UCMSCs (Pancreas), DT: T2DM rats treated with 0.2 ml of human UCMSCs (Tail vein), DI: T2DM rats treated with 0.2 ml of human UCMSCs (Intraperitoneal), DPA: T2DM rats treated with 0.2 ml of human UCMSCs (Pancreatic dorsal artery). (* $P<0.05$, ** $P<0.01$ and *** $P<0.001$ vs DM Group, with Cohen's $d > 0.8$ indicating a large effect size).

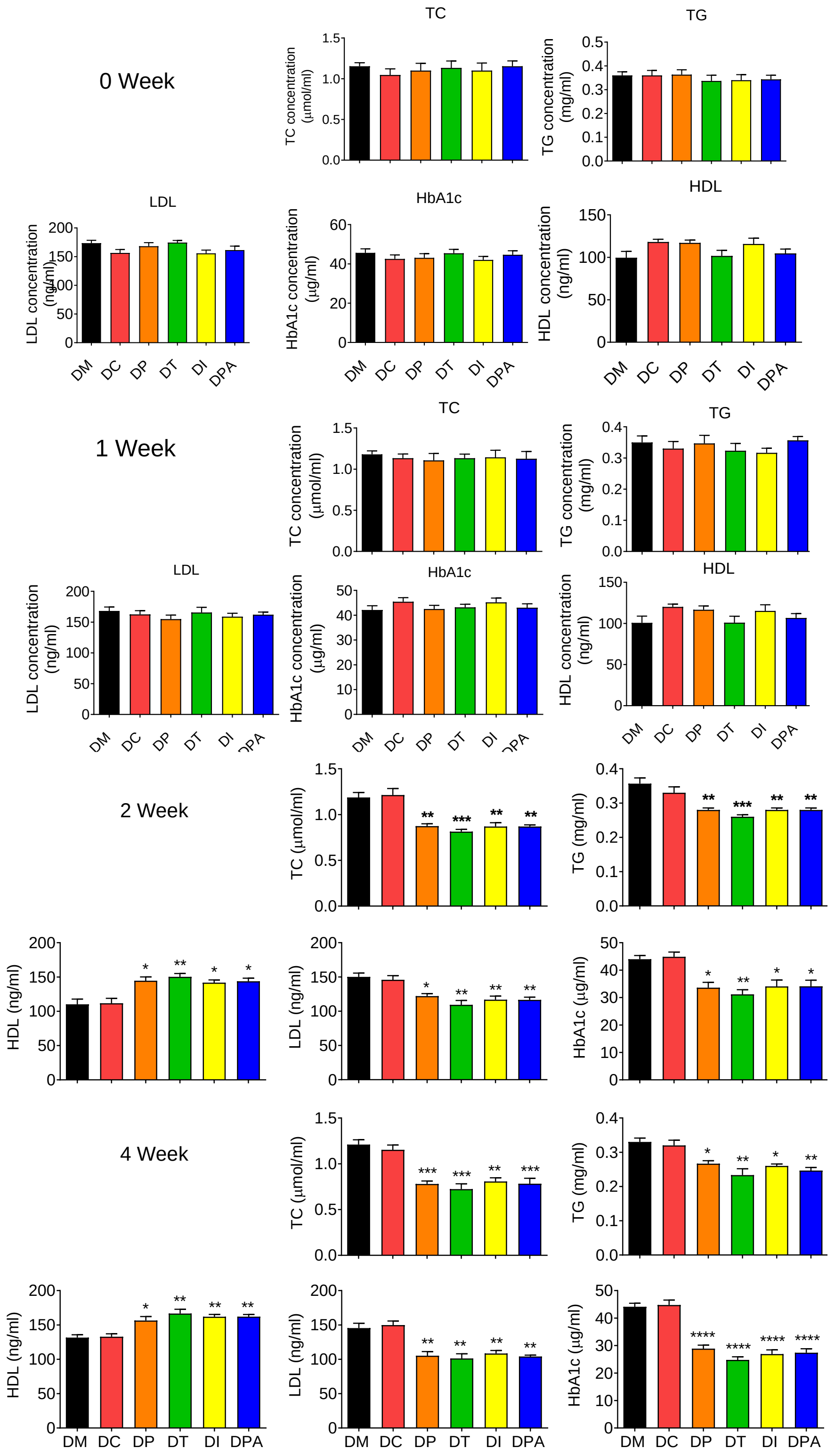

Figure S2. TC, TG, HDL, LDL, and HbA1c in serum concentrations after treatment in 0 to 4 weeks. DM: T2DM rats without treatment, DC: T2DM rats treated with 0.2 ml saline (Tail vein, negative control), DP: T2DM rats treated with 0.2 ml of human UCMSCs (Pancreas), DT: T2DM rats treated with 0.2 ml of human UCMSCs (Tail vein), DI: T2DM rats treated with 0.2 ml of human UCMSCs (Intraperitoneal), DPA: T2DM rats treated with 0.2 ml of human UCMSCs (Pancreatic dorsal artery). TC: Total cholesterol, TG: Triglycerides, HDL: High-density lipoprotein, LDL: Low-density lipoprotein, HbA1c: Hemoglobin A1c.

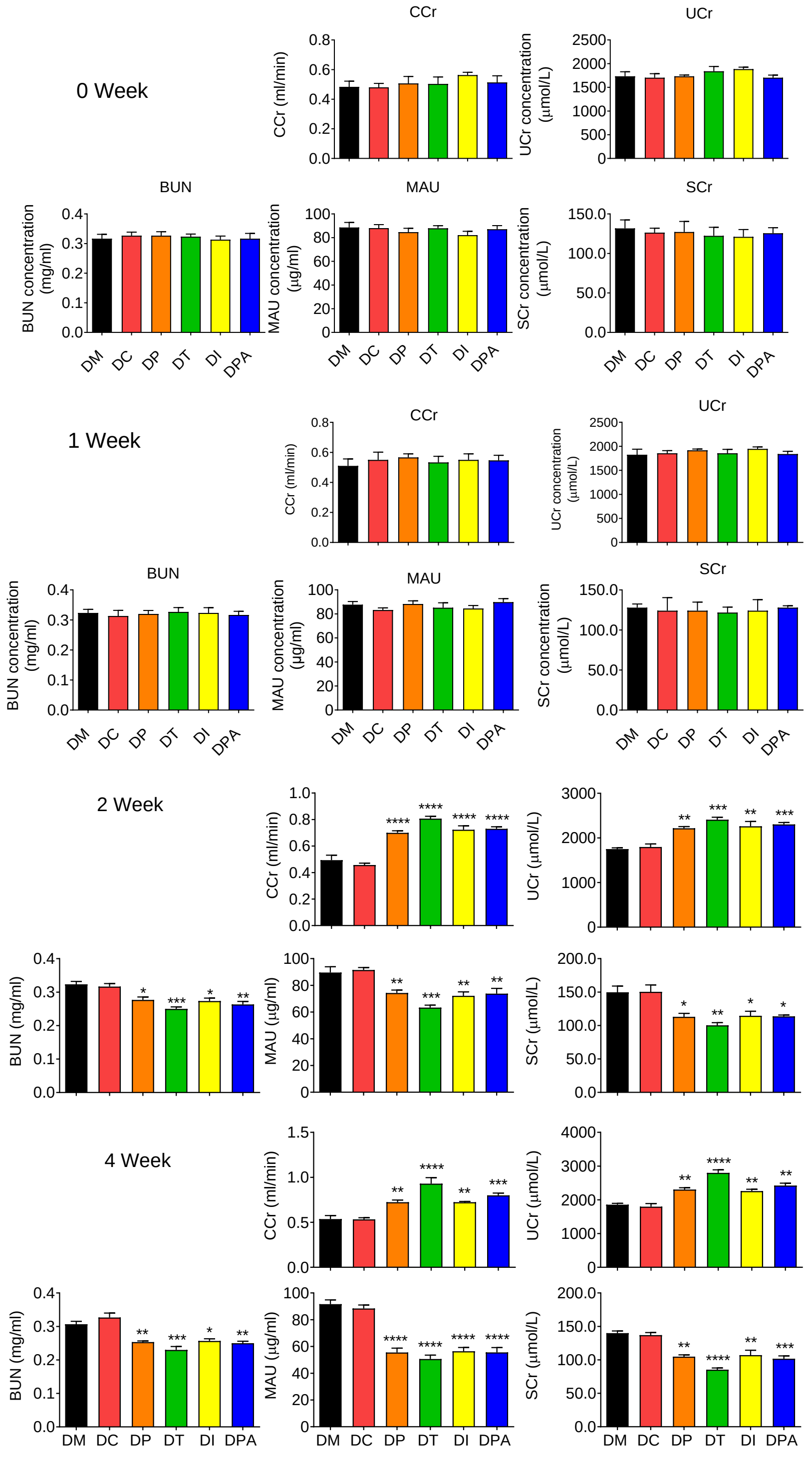

Figure S3. BUN, MAU, SCr, UCr and CCr in serum concentrations after treatment in 0 to 4 weeks. DM: T2DM rats without treatment, DC: T2DM rats treated with 0.2 ml saline (Tail vein, negative control), DP: T2DM rats treated with 0.2 ml of human UCMSCs (Pancreas), DT: T2DM rats treated with 0.2 ml of human UCMSCs (Tail vein), DI: T2DM rats treated with 0.2 ml of human UCMSCs (Intraperitoneal), DPA: T2DM rats treated with 0.2 ml of human UCMSCs (Pancreatic dorsal artery). BUN: Blood urea nitrogen, MAU: Microalbuminuria, SCr: Serum creatinine, UCr: Urinary creatinine, CCr: Creatinine clearance.

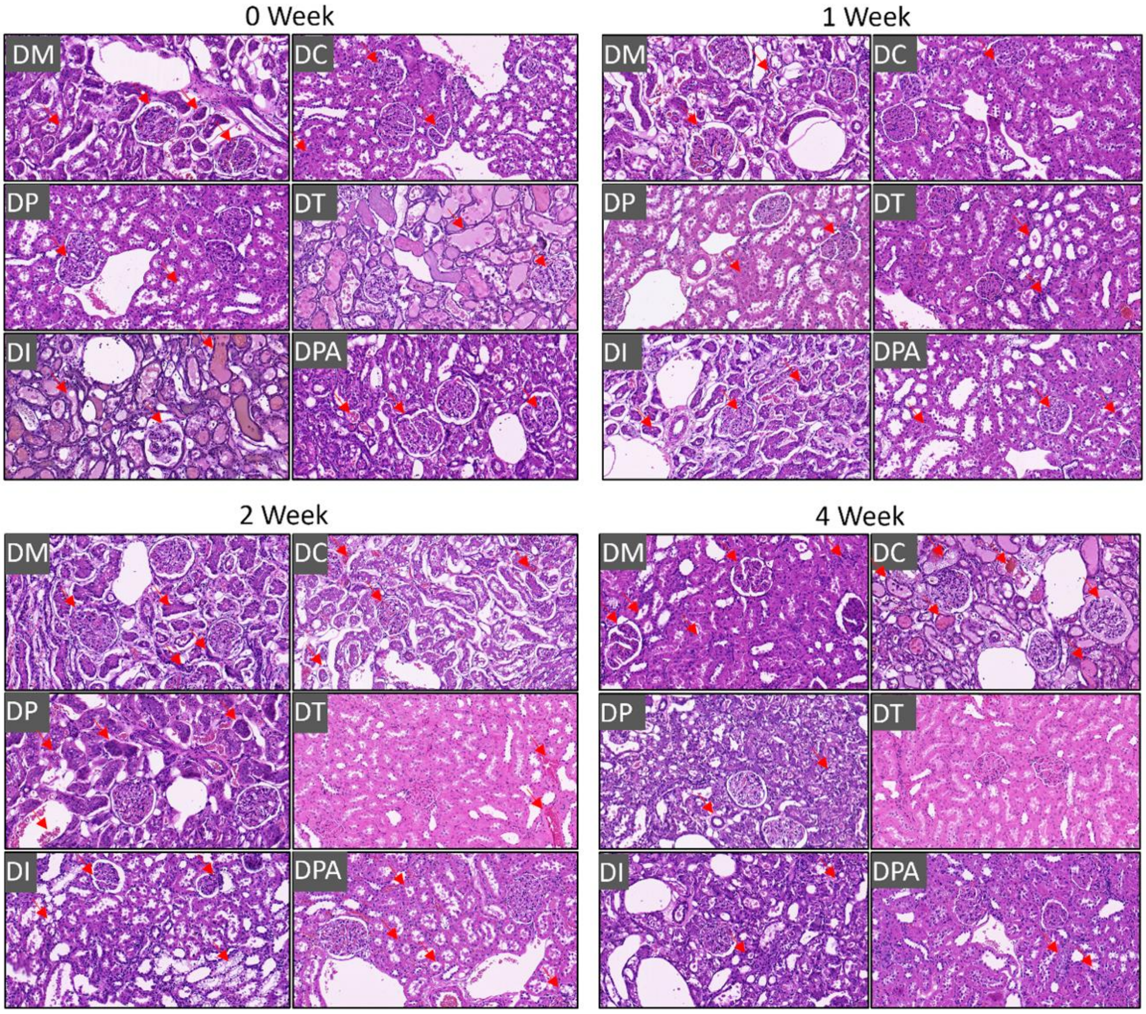

Figure S4. H&E staining of renal tissues (20×). Representative images show kidney structural alterations in different groups. The DM and DC groups exhibited renal corpuscular atrophy, tubular epithelial hypertrophy, irregular tubular lumen morphology, necrosis with calcium salt deposition, and vacuolar degeneration. In contrast, the DP, DI, and DPA groups showed progressive improvement, with the DI group demonstrating the most significant renal recovery by week 4, characterized by nearly normal glomeruli, restored tubular morphology, and minimal pathological alterations.

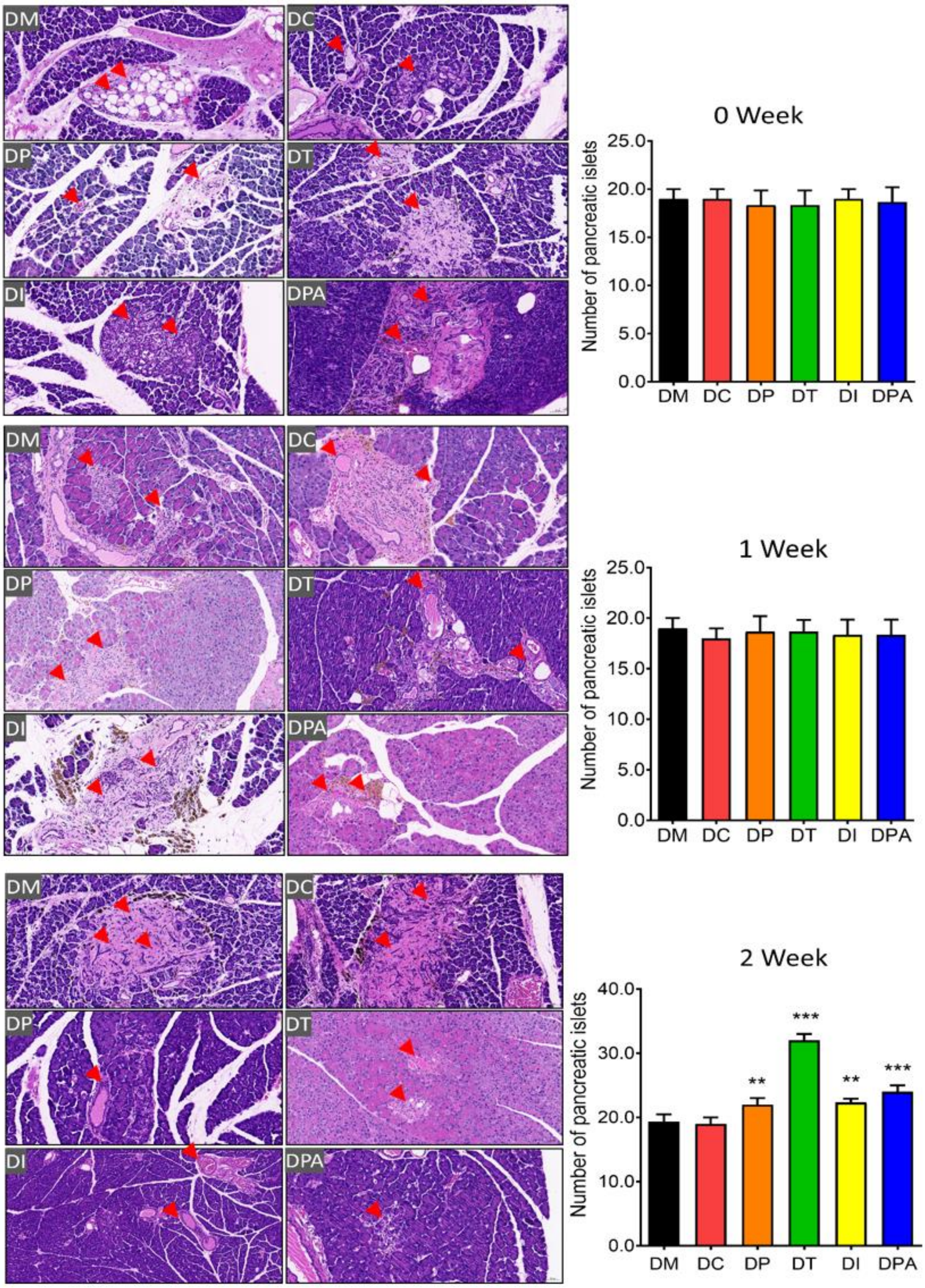

Figure S5. H&E staining of pancreatic tissues and the number of islets (20×) (** P<0.01, *** P<0.001, vs DM Group with Cohen's d > 0.8 indicating a large effect size). Severe pancreatic tissue damage was present in the DM and DC groups, characterized by islet cell atrophy and focal vacuolar changes. By week 2, the number of islets significantly increased in the DP, DT, DI, and DPA groups compared to the DM and DC groups, with the DT group exhibiting the most pronounced increase.

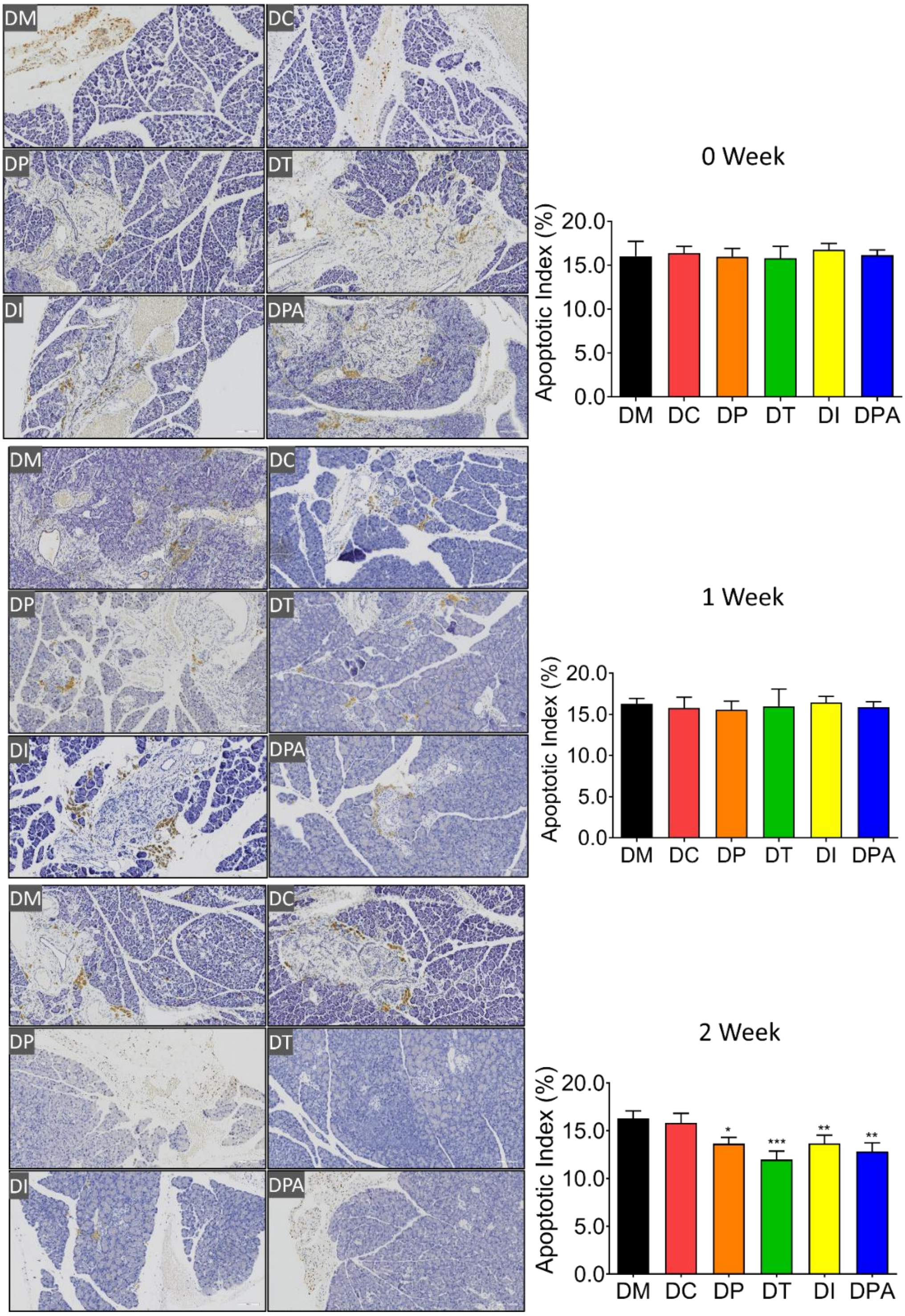

Figure S6. TUNEL staining of pancreatic tissues after 0, 1 and 2 weeks of treatment (20×) (*** P<0.001, **** P<0.0001 vs DM group, with Cohen's d > 0.8 indicating a large effect size).

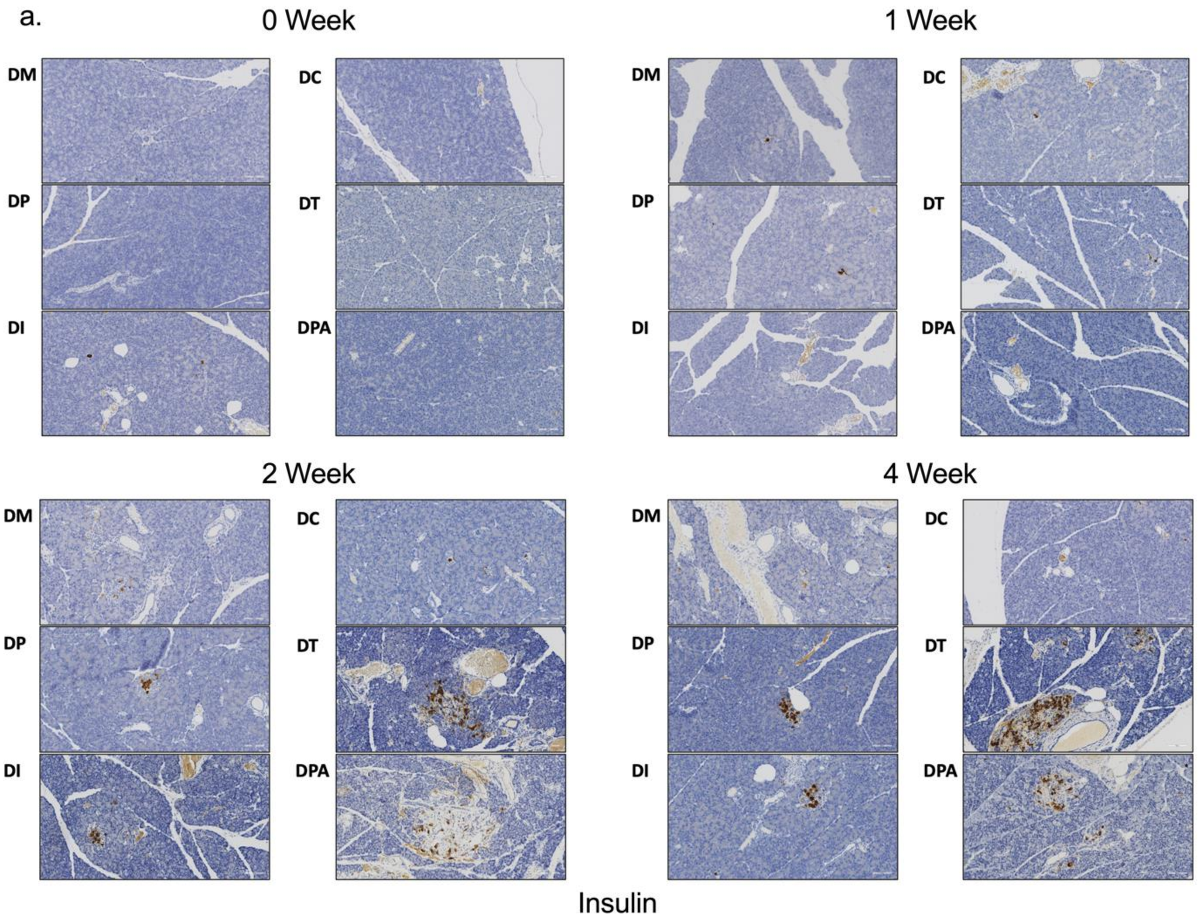
a.
0 Week
1 Week
2 Week
4 Week
DM
DC
DP
DT
DI
DPA
Insulin

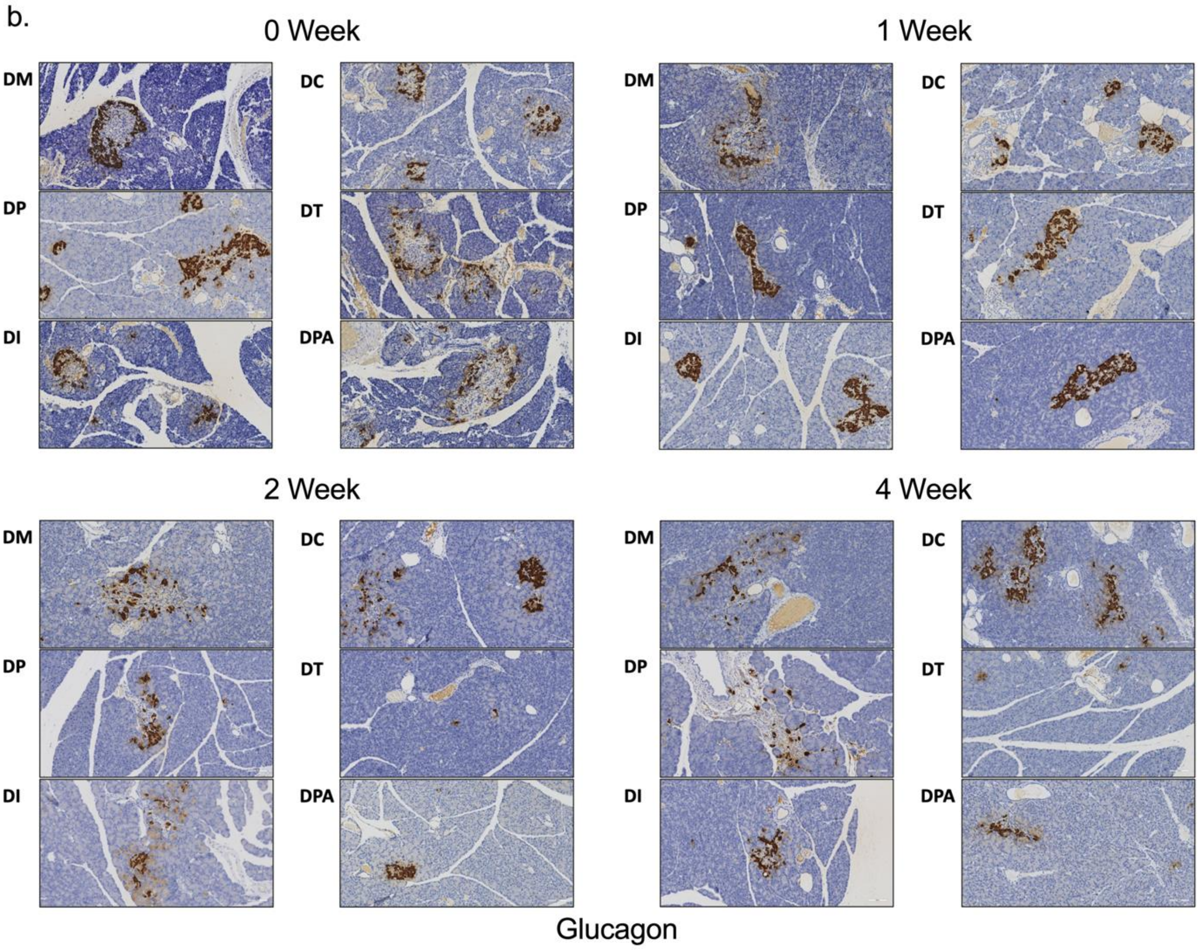
b.
0 Week
1 Week
2 Week
4 Week
DM
DC
DP
DT
DI
DPA
Glucagon

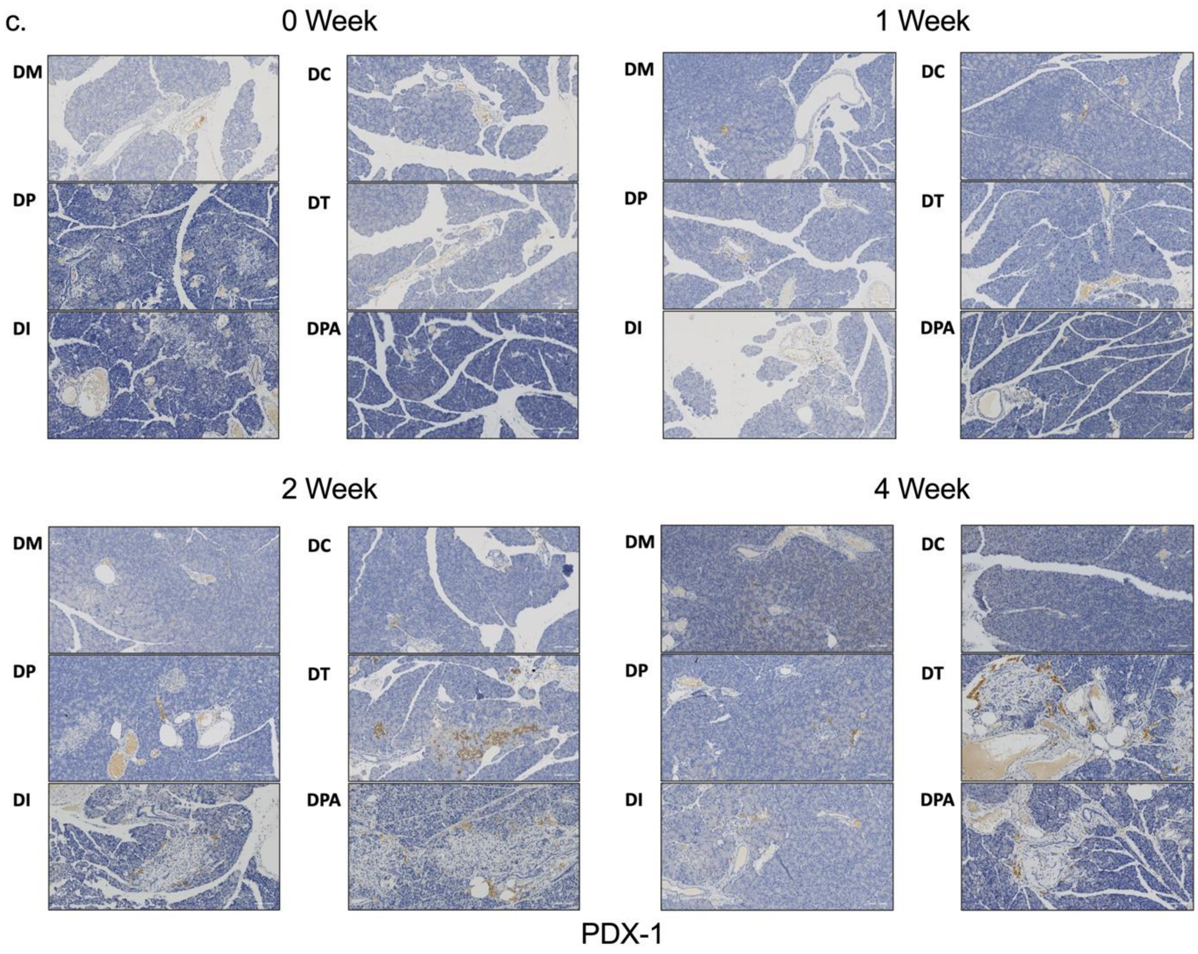
c.
0 Week
1 Week
DM
DC
DP
DT
DI
DPA
DM
DC
DP
DT
DI
DPA
2 Week
4 Week
DM
DC
DP
DT
DI
DPA
DM
DC
DP
DT
DI
DPA
PDX-1

d.

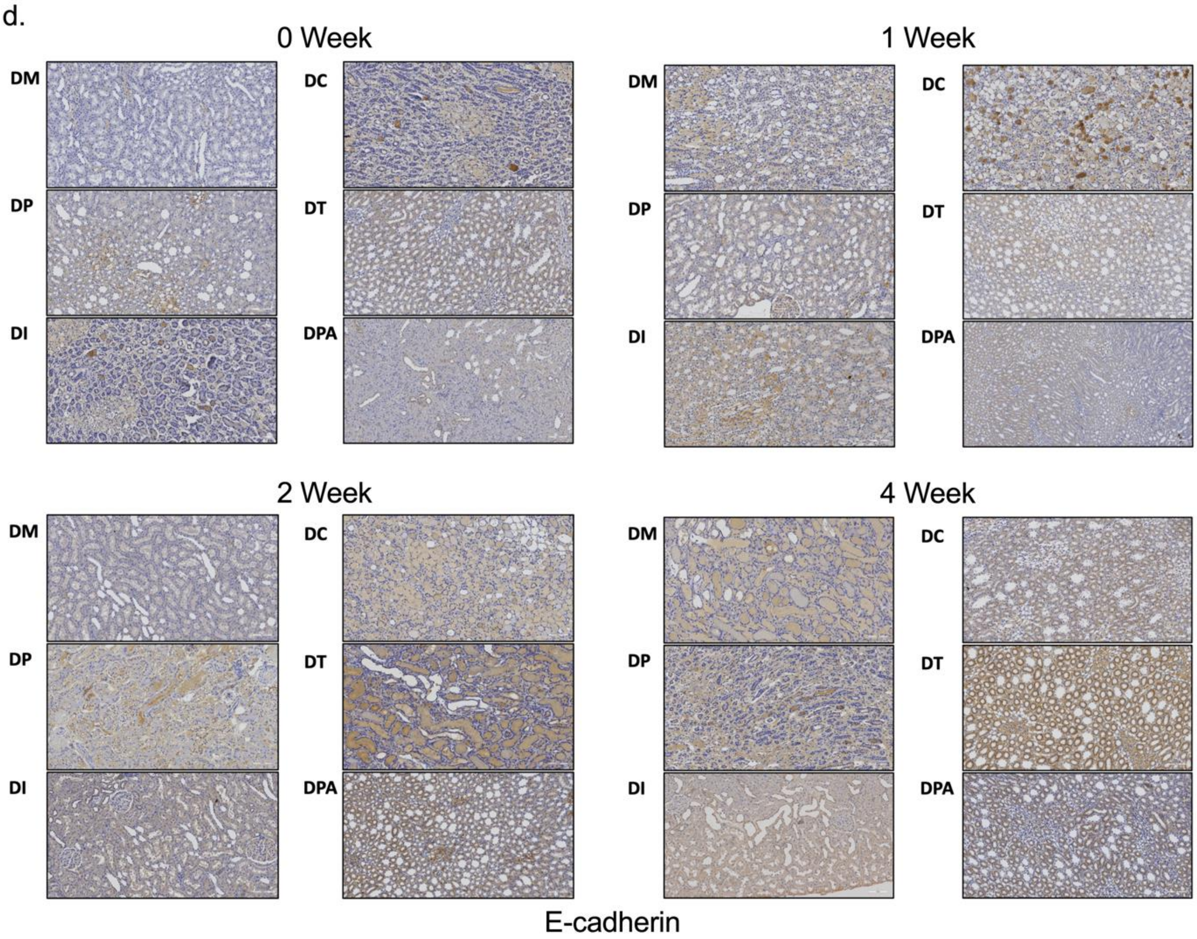
0 Week
DM
DC
DP
DT
DI
DPA
1 Week
DM
DC
DP
DT
DI
DPA
2 Week
DM
DC
DP
DT
DI
DPA
4 Week
DM
DC
DP
DT
DI
DPA
E-cadherin

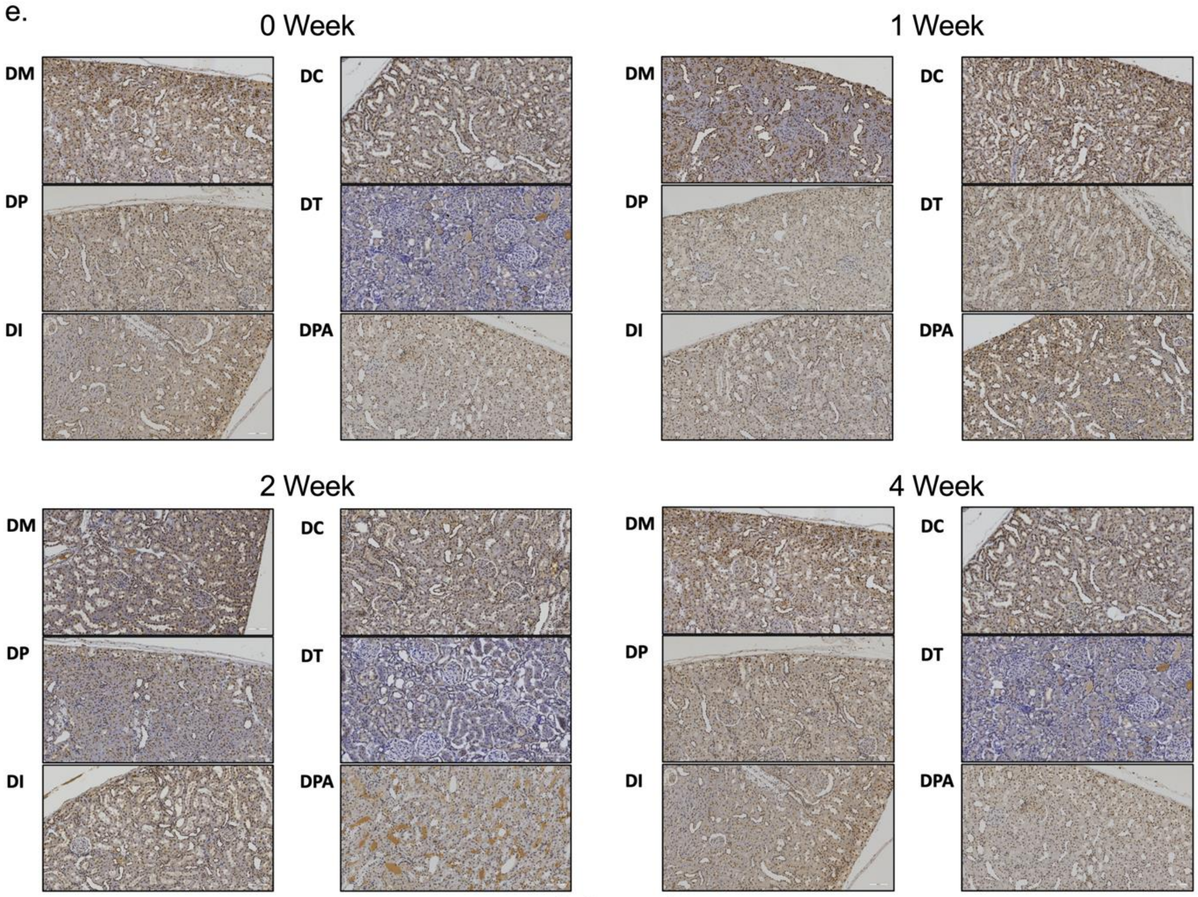

Collagen-I

f.

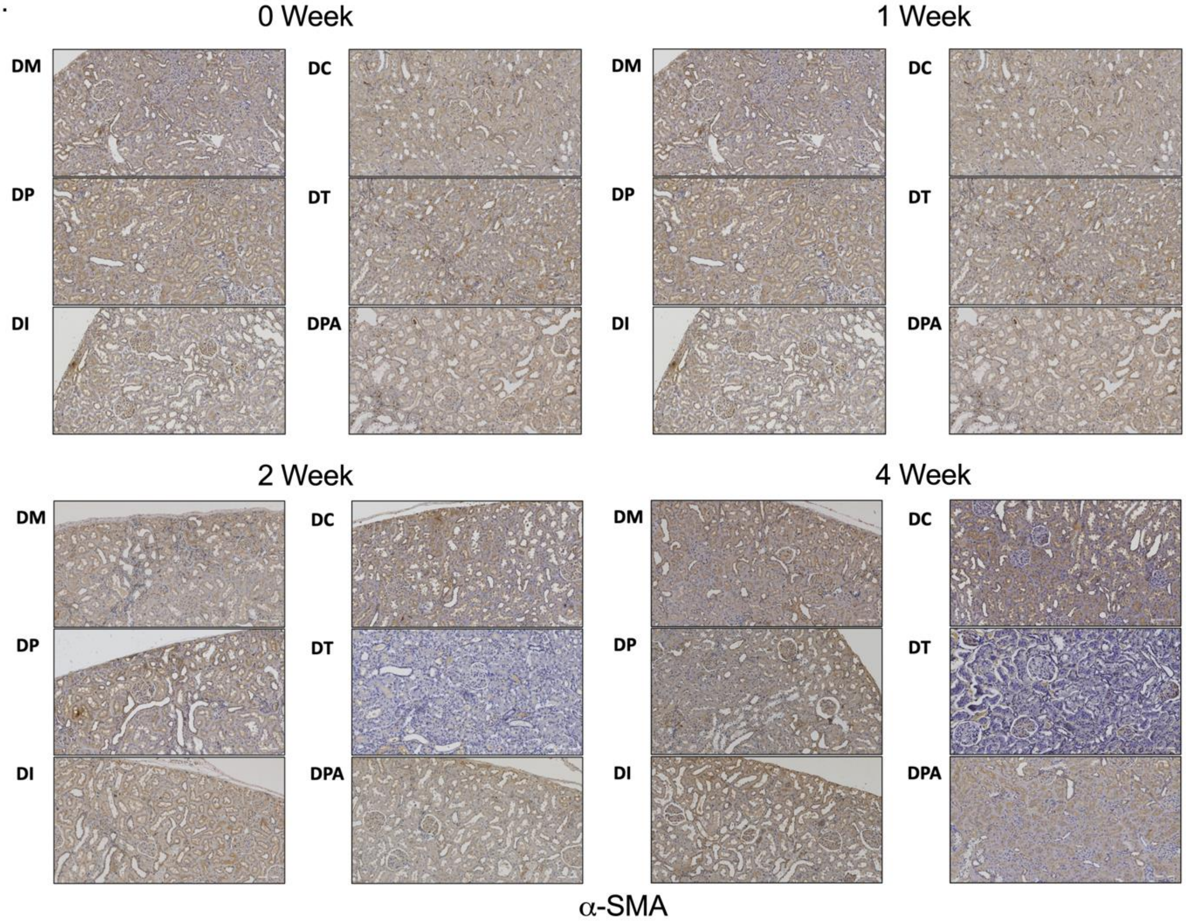

Figure S7. Immunohistochemistry staining of pancreatic tissues, **a**: Insulin, **b**: Glucagon, **c**: PDX-1, **d**: E-cadherin, **e**: Collagen-I, **f**: α-SMA (20×).

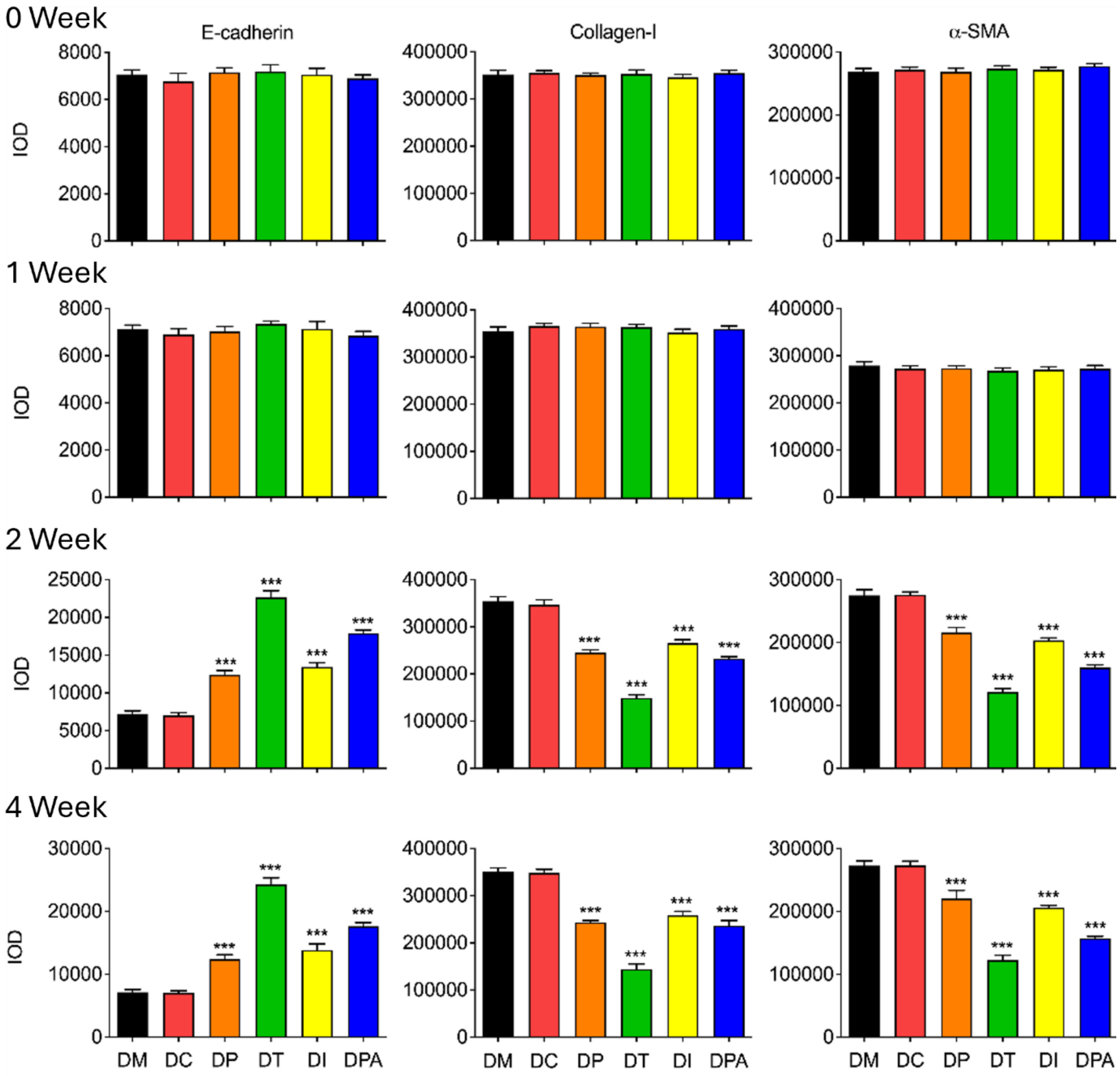

Figure S8. Expression of e-cadherin, collagen-I, and α-SMA in kidney after 0, 1, 2, and 4 weeks of human UCMSCs treatment (*** P<0.001 vs DM Group, with Cohen's d > 0.8 indicating a large effect size). IOD: Integrated optical density, α-SMA: α-smooth muscle actin.
